# Facteurs associes au port de charge céphalique chez des enfants au Bénin: étude transversale

**DOI:** 10.11604/pamj.2016.23.136.7502

**Published:** 2016-03-25

**Authors:** Barnabé Akplogan, Alain Mahoutin Hounmenou, Oscar Aze, Sakibou Essofa Alegbeh, Alain Azondekon

**Affiliations:** 1 Laboratoire de Biomécanique et Performance (LABIOP), Institut National de la Jeunesse, de l'Education Physique et du Sport (INJEPS), Université d'Abomey-Calavi, Bénin; 2 Laboratoire de Physiologie de l'Exercice (LPE), 10 rue de la Marandière 42270 Saint Priest en Jarez Saint Etienne/Université Jean Monnet de Saint Etienne, France; 3 Unité de Recherche de l'Hôpital d'Instruction des Armées de Cotonou, Bénin

**Keywords:** Port de charge céphalique, facteurs associés, enfants

## Abstract

**Introduction:**

Le port de charge céphalique par les enfants est une méthode de manutention courante au Bénin. Peu d’étude sont investigué sur le port de charge céphalique chez les enfants.

**Méthodes:**

Cette étude transversale vise à faire l’état des lieux et à identifier les facteurs associés au port de charge céphalique chez des enfants au Bénin. Au total,300 enfants âgés de 13,7 ± 2,6 ans ont participé à l’étude dans les 12 départements du Bénin. La méthode non probabiliste et la technique accidentelle ont été utilisées pour déterminer la taille de l’échantillon. La masse portée par les enfants constitue la variable dépendante. L’âge, la taille, les sites corporels des douleurs, l'ancienneté dans le port de charge etla fréquence hebdomadaire du port céphalique de charge constituent les variables indépendantes.

**Résultats:**

Le rapport de la masse portée sur le poids corporel est évalué en moyenne à 66%. Pendant et après le port de charge, les douleurs ressenties sont localisées essentiellement au cou, au dos et au bas du dos. Le test de corrélation entre charge portée et la taille indique r = 0,58 (p < 0,001).

**Conclusion:**

Cette étude indique que les enfants surchargent leur rachis lors du port de charge céphalique.

## Introduction

Les populations des pays de l'Afrique subsaharienne recourent à différents modes de port de charge pour déplacer des objets. La plupart du temps, il s'agit de marchandises ou de matériels utilitaires lourds (bois de chauffe, jarres d'eau), transportés sur la tête en marchant [1, 2]. Le port de charge sur la tête fait donc partie intégrante de la vie sociale et économique en Afrique subsaharienne; c'est la conséquence de la pénurie d'infrastructures routières, de la rareté subséquente des moyens de transport, du coût élevé des transports et du niveau très bas du revenu mensuel des ménages. Ce mode de portage est couramment utilisé parce que peu onéreux [3]. Lors du port de charge sur la tête, c'est sur la colonne vertébrale, structure principale que se repose la quasi-totalité de la charge portée [4]. Le squelette axial subit des contraintes considérables conduisant à l'altération discale [5, 6]. Les conséquences du portage céphalique sur la colonne vertébrale sont diverses et variées: déformations (scoliose, lordose, cyphose), tassement des vertèbres [7]. A cela, il faut ajouter les lésions sévères affectant le rachis thoracique et lombaire pouvant compromettre le pronostic fonctionnel et parfois vital [8]. Le port de charge céphalique est un mode de portage auquel toute les couches sociales ont recourt et particulièrement les enfants [9]. Au Bénin, les études portant sur les conséquences du port de charge céphalique sur le rachis de la femme adulte en milieu rural ont révélé des microtraumatismes comme la cyphose, l'hyperlordose, la lombalgie et la hernie discale [10]. Les enfants étant en pleine croissance et la maturation osseuse en construction, il est possible qu'une exposition prolongée à cette pratique soit dangereuse et nuisible pour leur santé. Cette étude a pour but de faire un état des lieux du phénomène de port de charge céphalique chez les enfants au Bénin et d'identifier les facteurs de risque associés à ce type de portage.

## Méthodes

### Type d’étude et cadre de réalisation

Il s'agit d'une étude transversale, réalisée de février à août 2013 au autour des marchés des villes et campagnes des 12 départements du Bénin.

### Population et échantillonnage

La population visée par l’étude est constituée des enfants béninois (garçons et filles) porteurs de charge céphalique. L’échantillon d’étude a été constitué par la méthode non probabiliste. Le choix des enfants a été effectué par la technique accidentelle (seuls les présents les jours de l'enquête et répondant aux critères d'inclusion ont été recrutés). Les critères d'inclusion dans l’échantillon sont les suivants: être âgés de 6 ans au moins et de 17 ans au plus; être porteur ambulant et régulier des charges sur la tête (quatre jours au moins de port par semaine); avoir donné son consentement volontaire et motivé (par un parent au moins); avoir deux (2) ans au moins d'ancienneté de port de charge. La taille minimale (n) de l’échantillon a été déterminée grâce à la formule de Schwartz [11], puis arrondie à une marge de 10%. Au total 300 sujets ont pris part à l’étude.

### Matériels et techniques

Les mesures de la masse corporelle, de la taille debout et de la distance parcourue ont été effectuées en utilisant respectivement un pèse personne BR 9012 (Camry, Chine), précis à 0,1 kg près; une toise murale 206 M (Seca-Bodymeter, France) graduée au millimètre près et des podomètres SW-800/801 (DIGI-WALKER, Japon). Une fiche d'enquête a servi au recueil des données.

### Collecte des données

L'enquête a été réalisée par deux personnes préalablement formées dans chaque région. L'une de ces personnes parle couramment la langue de la région cible. Il s'agit d'un enquêteur principal et d'un aide. Ces personnes ont communiqué avec les enfants dans leurs langues maternelles respectives (Fon, Idatcha, Dendi, Batonou, Adja, Minan, Nago et Wémè). Dans chaque localité, l'assistance des chefs de quartiers a été requise pour faciliter la mobilisation des enfants. Les mesures de la masse corporelle et de la masse portée ont été réalisées chez les enfants pieds nus dans la tenue qu'ils ont portée. La mesure de la taille debout a été effectuée entre le plan des pieds nus et le sommet de la tête en position debout.

### Variables étudiées

#### Variable dépendante

Dans cette étude, la masse portée par chaque enfant représente la variable dépendante. Elle est opérationnalisée en deux modalités (charge portée < 20% de la masse corporelle et charge portée ≥ 20% de la masse corporelle), elle est déterminée par la formule suivante: **Masse portée** (kg) = masse corporelle avec charge - masse corporelle sans charge.

#### Variables indépendantes

Les variables indépendantes sont: l’âge calendaire, la taille debout, l'ancienneté dans le port céphalique (nombre d'années de port de charge), à deux modalités: ancienneté < 2 ans et ancienneté ≥ 2 ans; la distance parcourue à pieds avec charge (< 2 km ou ≥ 2 km); la fréquence hebdomadaire du port céphalique de charge (< 3 jours ou ≥ 3 jours); troubles musculo-squelettiques appréciés selon que l'enfant ressent ou non des douleurs au corps pendant ou après le port de charges sur la tête; localisation des sites de douleurs (le cou, le dos, le bas du dos et autres parties corporelles).

### Analyse statistique

Les données ont été analysées à l'aide du logiciel SPSS (version 20). La description des variables a été faite en utilisant des proportions pour les variables catégorielles, la moyenne ± écart type pour les variables quantitatives de distribution normale. La normalité de la distribution a été étudiée par le test de kolmogorov-Smirnov. La corrélation entre la charge portée, l’âge, la masse corporelle et la taille a été étudiée en utilisant le diagramme de corrélation et le test de corrélation de Bravais Pearson. La comparaison des médianes a été faite par le test de Mann Whitney. Le niveau de signification des tests statistiques a été fixé à p < 0,05.

### Considérations éthiques

L’étude a reçu l'approbation du Comité Scientifique Sectoriel des Sciences et Techniques des Activités Physiques et Sportives (CSS/STAPS) de l'Institut National de la Jeunesse, de l'Education Physique et du Sport (INJEPS). Tous les parents des enfants qui ont participé à cette étude ont d'abord été informés dans leur langue maternelle des objectifs poursuivis, des mesures à effectuer, du déroulement de l'enquête avant de donner leur consentement éclairé et écrit. Le consentement pour que les enfants participent à l’étude a été donné par les parents. Néanmoins l′accord volontaire et non-coercitif a été verbalement obtenu chez chaque enfant.

## Résultats

### Les caractéristiques socio démographiques et biométriques

**Figure 1:**
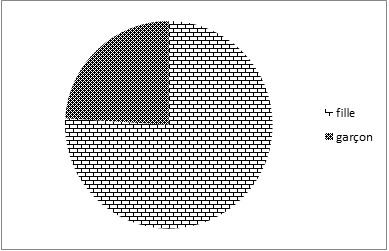
Répartition selon le sexe

**Figure 2:**
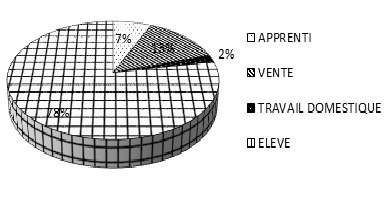
Occupation des sujets

L’échantillon d’étude est composé de 300 enfants (Figure 1) avec une prédominance de filles 76% pour 24% de garçons (228 filles et 72 garçons recrutés dans les 12 départements du Bénin). La taille moyenne des sujets est de 145 cm avec une masse corporelle moyenne de 40kg. L’âge des enfants varie de 6 ans à 17 ans avec une moyenne de 13,06. La Répartition des enfants selon leur occupation indique que 6,7% sont des apprentis, 13,3% sont des vendeurs ambulants, 78% des élèves ou écoliers et 2% des ménagères (Figure 2).

### Le port de charge céphalique

**Figure 3:**
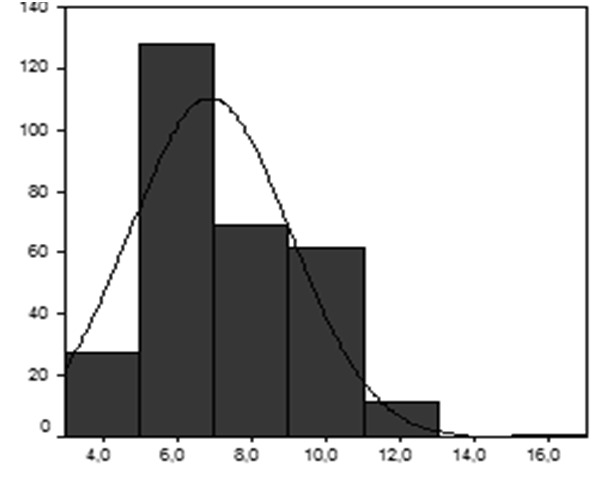
Répartition selon l’âge au début du portage

L’âge de début de port de charge selon les enfants enquêtés varie entre 3 et 13 ans avec une moyenne de 6 ± 2,6 ans dans le groupe étudié (Figure 3).

### Les troubles musculo-squelettiques liés au port de charge céphalique

**Figure 4:**
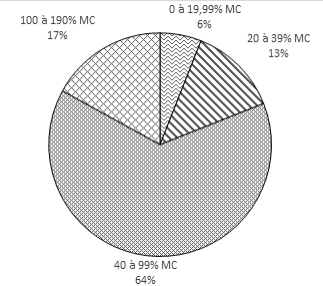
Catégorisation de la masse portée chez les enfants (n= 300)

Le trouble musculo-squelettique le plus signalé par les enfants est la douleur spinale. Cette douleur est identifiée depuis 2 ans au moins chez les sujets. Certains enfants la supportent depuis 9 ans. Pendant et après le port de charge sur la tête, les douleurs sont localisées essentiellement au cou, au dos et au bas de dos. Les douleurs identifiées chez les enfants sont localisées essentiellement dans les régions cervicales, dorsales et lombaires que les autres régions corporelles selon les propos des enfants (Tableau 1). L’étude révèle que 6% des enfants portent des charges inférieures à 20% de leur masse corporelle; 13% portent des charges variant entre 20% et 40% de leur masse corporelle; 64% transportent des charges entre 40% et 100% de leur masse corporelle et 17% des sujets surchargent leur rachis à plus de 100% de leur masse corporelle (Figure 4).

**Tableau 1:** Sites de la douleur déclarée par les enfants enquêtés (n=300)

	Pendant le port de charge Pourcentage (%)	Après le port de charge Pourcentage (%)
Tête	50	31
Cou	84,33	69
Dos et bas du dos	60	45
Autres sites	29,66	13

### Association entre la charge portée et le sexe

**Figure 5:**
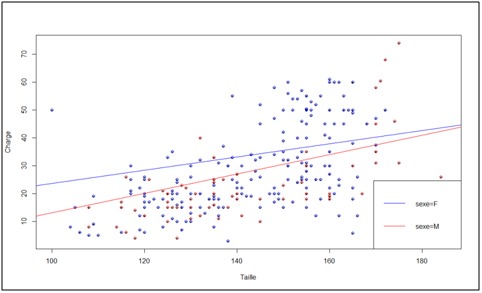
Association entre charge portée et la taille

La Figure 5 montre l'association entre la charge portée et la taille. Cette association montre que la taille est moyennement corrélée avec la charge portée r = 0,58 (p < 0,001).

## Discussion

### Les caractéristiques sociodémographiques et biométriques des enfants

Dans cette étude, l’âge des enfants enquêtés varie entre 6 et 17 ans. Suivant la Convention No 182 adoptée en 1989, il s'agit effectivement d'une population d'enfants. Elle est composée de 76% de jeunes filles contre 23% de garçons. Cet écart entre les deux sexes pourrait s'expliquer par le fait que les travaux domestiques et le port de charge céphalique sont plus confiés aux femmes et aux filles dans l'environnement traditionnel africain [12]. Par ailleurs, les sujets d’étude sont de jeunes enfants pubères en pleine croissance et âgés en moyenne de 13,06 ± 2,6 ans dont 78% sont des élèves. Le port de charge sur la tête est bien ancré dans les pratiques culturelles au Bénin [3, 10].

### Le port de charge céphalique

La masse de charge portée est estimée à 66% de MC est au-delà des données de la littérature qui indiquent que la masse maximale que peut porter un enfant sans risques nuisibles pour son organisme est moins de 20% de sa masse corporelle [13, 14]. Ce travail de recherche justifie les études qui stipulent que le port excessif de charge induit des effets délétères sur la santé des enfants. En effet, les enfants ont mentionnés lors de l’étude des douleurs cervicale, dorsale et lombaire. La masse excessive portée par les enfants pourrait être en relation avec la recherche du gain financier [3]. Ces parents évoquent essentiellement des raisons initiatiques et économiques pour justifier les charges imposées à leurs progénitures. L'initiation en Afrique subsaharienne se fait par imitation et contrainte. L'enfant est obligé de se soumettre à la volonté des parents et de les aider au cours de ses temps libres dans les activités génératrices de revenus.

### Les troubles musculo-squelettiques liés au port de charge céphalique

La douleur spinale est le trouble musculo-squelettique le plus signalé par les enfants. Cette douleur est ressentie chez les enfants depuis 2 ans au moins. Certains enfants la supportent depuis 9 ans. Pendant et après le port de charge sur la tête, les douleurs sont localisées essentiellement au niveau des cous, des dos et du bas du dos. Ces résultats sont confirmés par les travaux de Geereet al. qui ont prospecté sur les conséquences du transport de l'eau sur la tête sur la santé des enfants en Afrique du sud [15]. Ils ont abouti au résultat selon lequel la prévalence de la douleur au niveau de la colonne cervicale est très élevée. Ces troubles musculo-squelettiques constituent également des affections fréquentes dans la population des enfants porteurs de sac à dos [16]. Le port de charge génère en général chez les enfants des douleurs rachidiennes et fait donc partie du point de vue épidémiologique des facteurs de risque de la douleur spinale associés au mal de dos chez les adolescents [17].

### Association entre charge portée, la taille et le sexe

Les parents ne tiennent compte ni de l’âge ni de la masse corporelle des enfants pour les soumettre au port de charge céphalique. Par contre l'association entre la charge portée et la taille est moyennement corrélée r = 0,58 (p < 0,001). Les parents associent alors la capacité de l'enfant à porter plus de lourde charge à sa taille qu’à d'autres paramètres. Dans ces conditions, plus l'enfant est grand de taille, plus il est capable de porter de lourdes charges sur la tête. Cette pratique peut être dangereuse car susceptible de générer des effets délétères sur la colonne vertébrale de l'enfant.

## Conclusion

Cette étude vise à faire l’état des lieux et à identifier les facteurs de risque associés au port de charge céphalique chez des enfants au Bénin. La plupart des sujets portent sur la tête une charge supérieure à 20% de leur masse corporelle dans le but d'aider leurs parents. Ceci a l'effet d'améliorer les ressources économiques de la famille. Cet état de chose induit des symptômes typiques de troubles musculo-squelettiques susceptibles de traumatisme rachidien. Les autorités politico-administratives et les organisations non gouvernementales de protection des enfants devraient veiller à sensibiliser la population sur la diminution des charges afin d’éviter les conséquences délétères à long terme sur la santé des enfants.

### Etat des connaissance sur le sujet

- Il est montré que le port de charge sur le dos et sur la tête des enfants induit des troubles musculo-squelettiques.
- Pour éviter cela, les enfants doivent porter au plus 20% de leur masse corporelle.

### Contribution de notre étude à la connaissance

- Cette étude révèle que la taille des enfants est le paramètre auquel les parents se réfèrent pour leur faire porter des charges sur la tête.
- Plus l'enfant est grand mieux il est chargé.
- Cette pratique qui ne répond à aucune norme a des conséquences néfastes sur leurs rachis.
